# Biomedical Applications of Electrospun Nanofibers: Drug and Nanoparticle Delivery

**DOI:** 10.3390/pharmaceutics11010005

**Published:** 2018-12-24

**Authors:** Rajan Sharma Bhattarai, Rinda Devi Bachu, Sai H. S. Boddu, Sarit Bhaduri

**Affiliations:** 1 College of Pharmacy and Pharmaceutical Sciences, The University of Toledo Health Science Campus, Toledo, OH 43614, USA; RajanSharma.Bhattarai@rockets.utoledo.edu (R.S.B.); RindaDevi.Bachu@rockets.utoledo.edu (R.D.B.); 2 Department of Pharmaceutical Sciences, College of Pharmacy and Health Sciences, Ajman University, Ajman 2758, UAE; 3 Department of Mechanical, Industrial and Manufacturing Engineering, University of Toledo, Toledo, OH 43614, USA; Sarit.Bhaduri@utoledo.edu; 4 Department of Surgery (Dentistry), University of Toledo, Toledo, OH 43614, USA

**Keywords:** electrospinning, parameters, drug delivery, applications

## Abstract

The electrospinning process has gained popularity due to its ease of use, simplicity and diverse applications. The properties of electrospun fibers can be controlled by modifying either process variables (e.g., applied voltage, solution flow rate, and distance between charged capillary and collector) or polymeric solution properties (e.g., concentration, molecular weight, viscosity, surface tension, solvent volatility, conductivity, and surface charge density). However, many variables affecting electrospinning are interdependent. An optimized electrospinning process is one in which these parameters remain constant and continuously produce nanofibers consistent in physicochemical properties. In addition, nozzle configurations, such as single nozzle, coaxial, multi-jet electrospinning, have an impact on the fiber characteristics. The polymeric solution could be aqueous, a polymeric melt or an emulsion, which in turn leads to different types of nanofiber formation. Nanofiber properties can also be modified by polarity inversion and by varying the collector design. The active moiety is incorporated into polymeric fibers by blending, surface modification or emulsion formation. The nanofibers can be further modified to deliver multiple drugs, and multilayer polymer coating allows sustained release of the incorporated active moiety. Electrospun nanofibers prepared from polymers are used to deliver antibiotic and anticancer agents, DNA, RNA, proteins and growth factors. This review provides a compilation of studies involving the use of electrospun fibers in biomedical applications with emphasis on nanoparticle-impregnated nanofibers.

## 1. Electrospinning and Its History

Electrospinning is a process of forming micro/nanometer-sized polymeric fibers, either hollow or solid, with the application of the electric force on the polymeric solution at the tip of a conducting tube. It is one of the most commonly used techniques to obtain continuous fibers in the nanometer size range [1,2,3]. Electrospinning, also known as electrostatic spinning, has been used extensively for over three decades, and its usefulness in the fields of science and technology is still on the increase. Bose et al. first described the aerosols generated by the application of electric potential to the fluids in 1745 [4]. Further, Lord Rayleigh studied the amount of charge needed by the fluid to overcome the surface tension of a drop. Cooley and Morton patented the first device to spray the liquids under the influence of electrical charge in 1902 and 1903, and the fabrication of artificial silk was undertaken by Kiyohito et al. in 1929 [4]. Studies in the 1940s, 1950s and 1960s were limited and mainly focused on obtaining uniform-sized particle/fibers, decreasing the size, understanding and optimizing parameters, and designing the instruments [5,6]. In the 1990s, the process was finally taken up by educational institutions and, since then, many studies have been carried out on the versatility of manufacturing and the applications of the electrospun particles [2].

### 1.1. Process of Electrospinning

**Figure 1:**
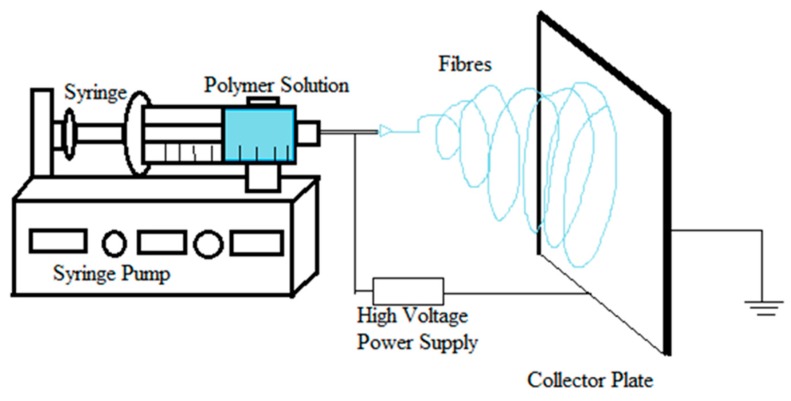
Schematic of electrospinning system. The system consists of polymer solution/melt in a syringe, mounted on a syringe pump, operating at a constant slow speed. High voltage direct current supply is connected to the needle of the syringe to charge the fluid. With sufficient voltage, the fluid forms a Taylor cone and then jet erupts from the cone towards the collector plate with whipping instability.

The electrospinning process involves the use of a very high voltage source (of either positive or negative polarity) to charge the polymer solution or melt, a grounded collector, and a syringe pump (Figure 1). It is advisable to perform the electrospinning process in a closed hood with minimal atmospheric influence, which serves as a safety measure for fibers and for the personnel. When a sufficient repulsive charge is accumulated and the repulsive force is equal to the surface tension, the drop surface on the conducting tube starts to form a cone called a Taylor cone. The conducting polymer solution/melt can exist in an equilibrium cone form under the influence of the electric field at an angle of 49.3° [7]. If the electric field is increased further, the repulsive force overcomes the surface tension. This results in the formation of a liquid jet from the Taylor cone when there is sufficient attraction between the molecules in the solution/melt. If the solution does not have sufficient cohesive attraction, the jets break and the resulting particles are sprayed onto a collector plate. The fiber originating from the Taylor cone travels through the air towards the collector plate, and during the process, the solvent evaporates, leading to a solid fiber deposit onto the collector plate [6,7]. The jet starts to experience instability after traveling through the air for a short distance and then starts to whip, thus increasing the path distance to the collector. This process assists in fiber thinning and solvent evaporation. There are multiple theories proposed on the reason behind jet instability. Some of the prominent theories include: repulsive interaction of charges in the polymer jet [8]; increase in charge density during jet thinning, thus increasing radial charge repulsion to cause jet splitting at a critical charge density [9]; and, “whipping” instability (spiraling loops) [10], causing the fiber to turn, bend [11] and/or splay [12]. Shin et al. have suggested the whipping stability concept based on the results obtained using high-speed photography, where a single strand of jet whipped very fast to give a cone-like appearance [11]. The “inverted cone” appearance had long been misunderstood as a splitting of the jet midway through its travel in the air.

### 1.2. Physics of Electrospinning

When the electric field/voltage is increased gradually and the surface of the drop becomes convex at a certain voltage, *V_c_* (critical voltage) is reached. At *V_c_*, the jets (electrospinning) and sprays (electrospraying) begin, which are represented by Equation (1):

(1) $$V_{c}=4\frac{H^{2}}{L^{2}}\;\left(Ln\frac{2L}{R}-\;\frac{3}{2}\right)\;\left(0.117\pi \gamma R\right)$$

where *H* is the separation between capillary and the collector, *L* is the length of capillary, *R* is the radius of capillary and *γ* is the surface tension [7]. This relationship was identified by Taylor, and a similar relationship for the potential required for the electrospraying of charged pendant drops of solutions from the pendant in a capillary tube was established by Hendrick et al., in Equation (2) [13,14]:

(2) $$V=300\sqrt{20\pi \gamma }r$$

where, *V* is the required voltage, *γ* is the surface tension and r is the radius of the pendant drop. Even though viscosity and conductivity are vital in the electrospinning process, they are missing from the equation. The use of applied voltage and the surface tension gives a representative equation for slightly conducting, medium- to low-conductivity solutions [14].

## 2. Parameters of Electrospinning

The electrospinning process is simple and does not involve heavy machinery. The processing parameters and solution parameters affect the size, porosity and uniformity of the fibers. These parameters have been discussed individually; however, the observations made in any particular study by a team of researchers are not universal. The modification of a parameter in one polymer may produce a totally different result with another polymer. In addition, none of the parameters act independently during the electrospinning process, and the final fibers are the result of a combination of several parameters.

### 2.1. Process-Related Parameters of Electrospinning

#### 2.1.1. Applied Voltage

**Figure 2:**
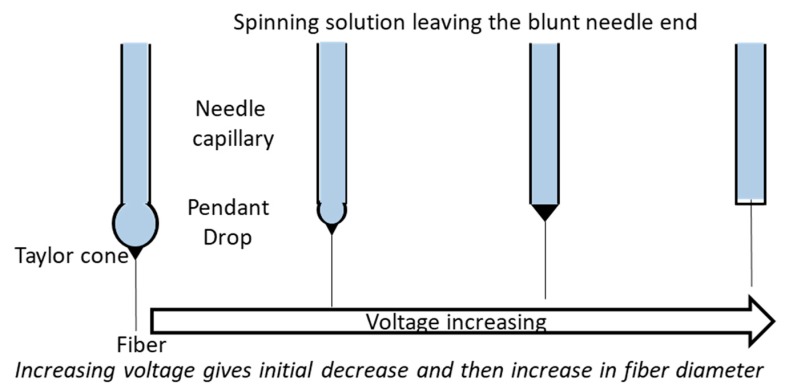
The effect of applied electric field on Taylor cone formation (dark colored tip). At low electric field, pendant drop is formed at the tip of the capillary and then a cone is formed on the tip. When the applied voltage is increased gradually, the drop size decreases until just a cone is formed at the tip of the capillary. If the voltage is further increased, fiber formation starts from within the needle without forming a visible Taylor cone on the blunt tip of the needle.

The primary factor influencing the formation of fibers is the strength of the applied DC voltage. The size of the fiber, formation of beads and absence of jet formation are all dependent on the applied DC voltage. With an increase in the applied voltage of a polyethylene oxide (PEO)/water system, the originating site for the jet changes from the tip of the pendant drop to the tip of the capillary, and the volume of the pendant drop decreases gradually (Figure 2). When the fiber is formed from within the capillary, the bead defects in the fiber increase [6,15]. A reduction in voltage up to some range moves the splaying (instability) point towards the tip of the capillary (i.e., the jet becomes unstable earlier) [12]. A study by Reneker and Chun reported no significant change in the fiber diameter with a change in the electric field with PEO solution [16]. On the other hand, electrospun polyvinyl alcohol (PVA)/water solution exhibited a broad diameter distribution above 10 kV [17,18]. In another study, Megelski et al. observed an increase in fiber size of polystyrene with a decrease in spinning voltage and with no significant change in the pore formation in fibers [19].

The fibers formed during the electrospinning process transport the charge to the grounded collector plate to close the circuit. This allows the electric current associated with the process to be measured; it is small (high voltage, 10–15 kV, high resistance and therefore very low current). When conductivity, dielectric constant and flow rate through the pump remain the same, an increase in current indicates an increase in the mass of the fibers formed. During the electrospinning process, the increase in current is gradual at first and then increases sharply from one voltage point, while sharp, steep increases are observed during electrospraying. The point of a sharp change in current could be indicative of the defect/change in the bead density [15].

Studies on the reversal of polarity obtained by grounding the solution in capillary and charging the collects resulted in inconsistent findings. Kilic et al. studied the effect of polarity on the electrospinning process of 7.5 wt% poly(vinyl alcohol)/water solution on production efficiency and nanofiber morphology. They concluded that due to the lack of columbic force acting on the polymer jet, the new reversed setup resulted in less nanofiber production. They also reported that the diameter and pore size of the web layer were much finer and more homogeneously distributed in the conventional setup [20]. On the other hand, Varesano et al. reported the production of good quality nanofibers with multi-jet electrospinning using both conventional and reverse polarity [21].

#### 2.1.2. Flow Rate

The polymer flow rate has a direct impact on the size, shape and porosity of electrospun fibers. A study on polystyrene/tetrahydrofuran (THF) solutions by Megelski et al. reported an increased fiber diameter and pore size with flow rate. However, at high flow rates, bead defects and flat, ribbon-like structures were observed due to insufficient drying [19]. In a different study, a similar effect on fiber morphology was observed with a 20 wt% solution of nylon 6 in formic acid at a constant electric field of 20 kV at flow rates of 0.1, 0.5, 1 and 1.5 mL/h. An optimal flow rate of 0.5 mL/h resulted in fibers with the narrowest fiber diameter distribution and a stable Taylor cone. However, at a flow rate of 0.1 mL/h, the Taylor cone could not be maintained and reduced over time to obtain fiber from within the capillary tip. At flow rates of 1.0 mL/h and 1.5 mL/h, the electric field was not sufficient to spin all the solution, and only a few drops were sprayed as they broke off from the capillary due to the gravitational force [22].

#### 2.1.3. Capillary–Collector Distance

The distance between the capillary and the collector is another factor which plays a significant role in controlling the size and morphology of the nanofibers [23]. This distance needs to be optimized as it might be the factor that distinguishes electrospraying and electrospinning. Typically a distance ranging from 10 to 20 cm is considered to be an effective spinning distance with the conventional method of electrospinning [24]. According to Doshi and Reneker, the larger the distance from the Taylor cone, the smaller the fiber diameter [9]. Jaeger et al. reported a decrease in fiber diameter (19 µm, 11 µm and 9 µm) with increasing distance (1 cm, 2 cm and 3.5 cm) from the orifice [12]. In another study investigating the electrospun polystyrene polymer fibers, decreasing the distance between the capillary and the collector from 35 cm to 30 cm did not change the diameter of the fibers significantly; however it led to the formation of non-homogeneous and elongated beads [19].

### 2.2. Solution-Related Parameters of Electrospinning

#### 2.2.1. Concentration of Solution

Viscosity and surface tension, which determine the spinnability of a solution, should be taken into consideration in determining the concentration of solution/melt for electrospinning. Surface tension is a dominating factor in a low concentration solution (low viscosity, <1 poise), and at such concentration, drops will be formed instead of a continuous fiber. At a higher concentration (viscosity >20 poise), the flow of the solution cannot be controlled and maintained. In the PEO/water study discussed above in Section 2.1.1, a concentration range of 4–10 wt% with viscosity and surface tension ranging between 1–20 poise and 55–35 dynes/cm, respectively, were studied. At low concentrations of 4%, fibers were not sufficiently dry and formed fiber junctions and bundles. At higher concentrations, straight and cylindrical fibers with fewer fiber junctions and bundles were reported. The diameter of the electrospun PEO fibers increased with concentration, and a bimodal size distribution was observed above 7 wt% concentration. The average diameter of the fibers was reported to be related to solution concentration through the power law relationship, with an exponent of 0.5 [15]. A statistical study by Sukigara et al. on regenerated silk proved that the silk concentration was the most important parameter in producing uniform fibers of a diameter less than 100 nm [25]. Fong et al. investigated the effect of viscosity on morphology defects (bead formation) of electrospun nanofibers formed from PEO solutions. An increase in bead diameter and decrease in bead density was observed with solution viscosity. At higher viscosity, the shape of beads changed from spherical to spindle resulting in the formation of nanofibers with diminished morphology defects [26]. Solutions with low polymer concentration and high surface tension produced droplets as the viscoelastic forces could not overcome the repulsive forces of charge, resulting in the fragmentation of fiber jet into droplets. At higher concentrations, the viscoelastic forces are sufficient to prevent the fragmentation resulting in the formation of smooth nanofibers. Nanofibers of polyacrylonitrile solutions were formed in the viscosity range of 1.7 to 215 centipoise. With increase in viscosity, the fiber jet length and the nanofiber diameter increased and the drop at the capillary tip changed from hemispherical to conical shape [27]. In a different study, 15 and 20% *w*/*v* solutions of poly(desaminotyrosyl-tyrosine ethyl ester carbonate) (poly(DTE carbonate)) were electrospun at a voltage of 10 kV to 25 kV at 10 cm distance. Beaded fibers were observed during electrospinning at a lower concentration solution until the voltage reached 20 kV, and the density of beads decreased until the voltage reached 15 kV; average fiber diameter increased, while increasing the voltage from 20 kV to 25 kV. At higher solution concentrations, the smooth fibers obtained showed an increasing diameter and decreasing fiber density with the increase in the electric field from 10 kV to 25 kV [28].

#### 2.2.2. Molecular Weight

The molecular weight of polymer influences the solution viscosity and thus has an important effect on fiber morphology. For example, decreasing the molecular weight of poly(vinyl alcohol) while maintaining other parameters constant resulted in the formation of bead-like structures. On the other hand, higher molecular weight resulted in smooth fibers initially followed by ribbon-like fibers upon further increase in molecular weight [29]. Ultra-high molecular weight polymers, such as polyacrylamide (molecular weight 9 × 10^6^ g/mol), exhibited a variety of fiber morphologies even with a minute change in the concentration within the range 0.3–3.0 wt%. Beaded and smooth fibers were formed at concentrations between 0.3 to 0.7 wt%, whereas smooth fibers with ribbons coexisted at 0.7 to 2 wt%. Above 2.0 wt%, only ribbons were formed, which were either helical or zigzag with triangular beads on them [30]. Additionally, a study on the melts of polypropylene with varying molecular weights showed an increase in the fiber diameter with molecular weight. High molecular weight polymers show the highest degree of entanglement and pose difficulties for the electric field to pull on individual polymer chains to obtain a thin fiber [31].

#### 2.2.3. Solution Viscosity

Viscosity determines the solution’s ability to form fibers. Smooth continuous fibers can be obtained at optimum viscosity for a particular polymer solvent combination. The viscosity, molecular weight of the polymer and polymer concentration are interrelated, and one cannot be independently judged. Low viscosity fails to form the fibers, while high viscosity requires a higher electric field for electrospinning, making it hard to operate [25,32]. Baumgarten observed the formation of fine droplets at a lower viscosity and with incomplete drying. At higher viscosity, the droplets bumped into each other in mid-air due to incomplete drying with acrylic polymer [27]. When a solution has low viscosity, surface tension dominates the process of fiber formation, but at optimum concentration, it is the combined effect of both parameters [29,33,34,35,36]. Yang et al. suggested a mixed solvent system of dimethylformamide (DMF) and ethanol (50:50) for obtaining the best electrospun fibers of poly(vinyl pyrrolidone) and attributed the success to the combined effect of solution viscosity and charge density [10].

#### 2.2.4. Surface Tension of the Solution

Surface tension is the measure of cohesive forces between the molecules in solution form and is dependent upon the solution composition, polymer and solvent(s) used. Yang et al. studied the influence of solvents on the formation of nanofibers with poly(vinyl pyrrolidone) and concluded that lower surface tension with high viscosity formed smooth nanofibers with ethanol as the solvent. They proposed the use of a multi-solvent system to obtain optimum surface tension and viscosity parameters for better electrospun fibers [10]. Like viscosity, surface tension can define the range of solvents and concentrations to be used in the electrospinning process [18].

#### 2.2.5. Conductivity and Surface Charge Density

The impact of conductivity and surface charge density of solution also plays an important role in the process of electrospinning. It is important to have high conductivity for a greater charge-carrying capacity. A highly conductive solution experiences a stronger tensile force in an electric field compared to the less conductive solution, making the former preferable for electrospinning. An increase in the conductivity of the solution causes a substantial decrease in the diameter of the nanofibers. The radius of the fiber jet varies inversely as the cube root of the conductivity of solution [37]. For preparation of acrylic microfibers, Baumgarten reported that the jet radius is dependent on the inverse cube root of electric conductivity [27]. Natural polymers, being polyelectrolytic, have better charge-carrying ability and lead to poor fiber formation compared to synthetic polymers [38]. Hayati et al. observed that stable jets could be obtained by semi-conducting liquids by applying sufficient voltage. Due to insufficient free charges, the insulating liquids, such as paraffin oil, could not build an electrostatic charge on the surface, while highly conducting water produced an unstable stream and sparking at higher electric fields. Consequently, insulating liquids and semiconducting liquids could produce stable fibers [39]. Yet another study on poly(vinyl alcohol) solution showed that the addition of a small amount of sodium chloride drastically increased the conductivity and decreased the fiber diameter [17]. The addition of sodium chloride has an effect on decreasing the occurrence of beads in PEO solution [26]. Sodium phosphate [38], potassium phosphate [38], ammonium chloride [26], and lithium chloride [26] are also used to obtain better fibers by changing the conductivity of solutions. Huang et al. used compounds soluble in organic solvents, such as pyridine, that react with formic acid in solution to form a salt. Pyridine improves the conductivity and can easily be removed to obtain dry fibers. The addition of 0.4 wt% of pyridine doubled the electrical conductivity of 2% nylon-4,6 in formic acid [40].

#### 2.2.6. Solvent Volatility

The distance between the tip of the capillary and the collector is only a few centimeters (typically 10–15 cm), and the path taken by the jet to reach the collector is a few folds more. The fibers formed during the process are porous and dry fast, depending on the choice of the solvent used for solubilization of the polymer. The insufficiently dried fiber may attach to itself mid-air [27], form ribbon-like fibers [19] or attach to itself after depositing on the collector. A study on polystyrene fibers showed that the use of more volatile tetrahydrofuran (THF) as a solvent produced high-density pores and increased the surface area of fibers by up to 40%. The same polymer in DMF lost the microtexture completely. A combination of these solvents in different ratios gave different morphology profiles as the volatility of the mixture varied [19].

## 3. Types of Electrospinning

The type of electrospinning process can have a significant impact on fiber formation in addition to the process and solution parameters. Two main aspects to deal with in the type of electrospinning are solution vs. melt electrospinning and nozzle configuration [6].

### 3.1. Solution vs. Melt vs. Emulsion Electrospinning

Electrospun fibers are generally obtained from polymer solutions or melts or emulsions. Melt spinning has high-throughput rate and process safety; solutions have the advantage of using a large variety of polymeric materials, lower energy consumption and superior mechanical, optical and electrical properties of prepared fibers. Emulsion electrospinning is needed for high melting polymers to prepare flame-retardant fibers. A comparison of different spinning methods using poly(lactic acid) (PLA) as a model polymer has been reported by Gupta et al. [41].

The melt of the polymer with other additives is extruded through the capillary, resulting in a thin fiber that cools and solidifies rapidly during its time in the air before depositing onto the collector [41]. Take-up speed, drawing temperature and draw ratio define the structural and tensile properties of the fiber. Draw ratio is the amount of stretching that the material undergoes during the drawing stage of electrospinning. The increase in draw ratio [42] and take-up speed [43] increases the molecular chain orientation along the fiber axis as well as the overall crystallinity of fibers formed from the melt. A higher entanglement of polymer fibers amongst themselves gives sub-par fibers compared to the solution of the same polymer.

The solution spinning method is ideal for polymers or blends of polymers that are thermally unstable or degrade upon melting. Based on how the solvent used for preparing the polymer solution is removed from the fibers, this method can be divided into two different types, namely, dry spinning and wet spinning. The dry spinning process uses hot air or inert gas on the polymer jet to facilitate the solvent’s evaporation and fiber solidification. For example, Postema et al. used a solvent combination of chloroform and toluene to produce PLA fibers of high strength by using dry spinning and hot-drawing processes. They were able to obtain an optimal tensile strength of 2.2 GPa by electrospinning at 25 °C [44]. The wet-spinning process involves the use of a viscous coagulation bath containing the liquid, which is miscible with the spinning solvent but not with the polymer. The interaction between the polymer solvent and non-solvent leads to phase separation and then solvent removal from the jet. This process is known to show fiber defects that can, to some extent, be limited by using 3–5 mm of air gap before the non-solvent, as this allows for stress relaxation of the polymeric chain [41].

In the emulsion electrospinning process, finely ground polymers, insoluble and non-melting, are mixed with another polymer solution with a catalyst and emulsifying agents. The formed emulsion is then electrospun by either the dry or the wet spinning method. This technique has been used in preparing fibers from fluorocarbons with high melting points, ceramics, and polymer blends with flame-retardant properties [45]. Persano et al. have discussed in detail the methods and their industrial application in their review [45].

### 3.2. Nozzle Configuration

**Figure 3:**
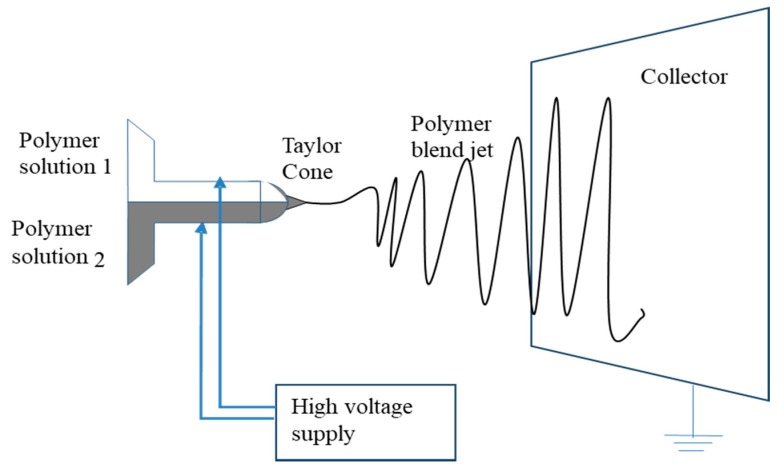
Side-by-side electrospinning schematic diagram: Polymer solutions 1 and 2 pass through separate capillaries, which are connected to the same high voltage supply, at either the same or different rates. A single Taylor cone is formed, which ejects the jet with non-uniform mixture of both the polymer solutions and, after drying, is deposited on the collector.

Nozzle configuration relates to the number and the arrangement of capillary tubes from which the jets of fibers emerge. A single nozzle configuration is the simplest and most common configuration, wherein the charged solution flows through a single capillary (Figure 1). This particular configuration has been used to electrospin different polymeric fibers either singly [46,47] or in combination [48] or solvent systems [10]. Co-electrospinning of polymer blends in the same solvent or a mixture was the first and most common modification in the process. Zhou et al. mixed polyaniline with poly(ethylene oxide) in chloroform to obtain nanofibers of a size below 30 nm [49]. Sometimes polymer blends are used to obtain the final properties of fibers. If the polymers are not miscible in a common solvent, or when a homogeneous solution of polymers cannot be obtained, thermodynamic and kinetic aspects should be considered for electrospinning. One way around that problem is to modify the nozzle configuration, where different polymer solutions are electrospun from different capillaries, side-by-side. In this type of configuration, two polymer solutions pass through separate capillaries arranged side-by-side, which are connected to a high voltage supply and never come into contact until they reach the end of the capillary. A single Taylor cone is formed, which ejects the jet with a non-uniform mixture of both polymer solutions and, after drying, is deposited in the collector (Figure 3). The side-by-side technique yields Janus fibers having two different materials on either side of the fiber. The special morphology obtained provides combinational properties of two different polymers and allows several post electrospinning modifications [50]. For this type of configuration to work, it is necessary that both the polymeric solutions have similar conductivity for them to form a single Taylor cone and eject as a mixture. A bicomponent system consisting of poly(vinyl chloride)/segmented polyurethane and poly(vinyl chloride)/poly(vinyl fluoride) was studied [51].

**Figure 4:**
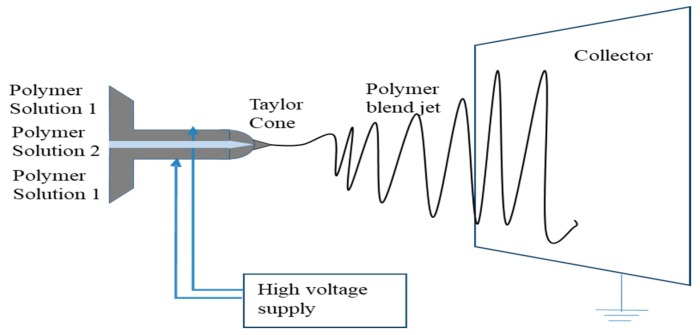
Coaxial electrospinning schematic diagram: Polymer solution 2 is passed through the inner capillary tube, while polymer solution 1 is passed through the outer capillary tube. The Taylor cone is formed where the inner solution (polymer solution 2) is surrounded by the outer solution. The jet erupts from the Taylor cone, and during that process, the polymer in the inner layer is coated with the polymer in the outer layer. The dried fiber with a core-shell design is then deposited on the collector.

A second type of nozzle configuration that has recently been introduced is coaxial configuration. Two separate polymer solutions flow through two different capillaries, where the small capillary is within the larger capillary. This configuration can easily encapsulate a small fiber within a larger fiber, forming core-shell morphology (Figure 4). In one study, living cells were encapsulated in poly(dimethylsiloxane) fiber with high cell viability (67.6 ± 1.9%) compared to the control cells (70.6 ± 5.0%). Though the initial viability was comparable, over time, issues with cell morphology and growth rate were observed. This was the first study of its kind, and authors noted the need for further research to obtain the best possible results for encapsulation of living cells [52]. Encapsulation of a model protein, fluorescein isothiocyanate conjugated bovine serum albumin (BSA), with poly(ethylene glycol) in poly(epsilon-caprolactone), was able to sustain the release of the drug, defining its use in drug delivery. Coaxial fibers of proteins, such as fibrinogen [53], BSA, and lysozyme [54] and growth factors, such as bone morphogenetic protein2 (BMP-2) [55], bFGF [56], PDGF [57], VEGF [58], and EGF [59] have been reported. Apart from the regular parameters that affect the quality of fibers being produced by coaxial electrospinning, the relative flow rate of the core and the shell solutions (ratios between 5:1 and 6:1) is important in determining the encapsulation efficiency [60]. At a lower flow rate, the core phase is not continuous, which results in breaking up of the core, whereas a higher flow rate causes the formation of pendent droplets. Wang and co-workers reported that at an optimal flow rate, the core diameter could be easily controlled. Also, the flow rate is related to the core diameter by scaling laws of d_f_~Q_c_^0.18^ where d_f_ represents the inner diameter of the fiber and Q_c_ represents the flow rate of core solution [61] This method is widely used in tissue engineering to achieve sustained, local and efficient gene and growth factor delivery to the cells [60,62,63]. Coaxial fibers of different non-steroidal anti-inflammatory drugs such as ketprofen [64], flurbiprofen axetil [65], and ibuprofen [64] and antibiotics such as levofloxacin, tetracycline hydrochloride, ciprofloxacin, moxifloxacin, and fusidic acid [66] were prepared and investigated. Though this method is now widely used, it still has the drawbacks of complexity of design and precise control of spinning parameters e.g., interfacial tension, viscoelasticity of the polymers or two different solutions used.

The multi-jet electrospinning based on multiple capillaries arranged in circular geometry is yet another modification of the nozzle that can both increase the throughput and facilitate scale up and commercialization. This modification increases the mat thickness, deposits in a larger area and can mix fibers of different materials for better strength and versatility of use [21,67]. In the study by Varesano et al., the morphology of PEO fibers was found to be acceptable in both polarities applied in standard and reverse configuration [21]. The main drawback of the multi-capillary method is the alteration of the electric field due to the presence of other electrospinning jets in the vicinity [68]. This can be overcome by using an auxiliary electrode of any polarity [69] or by using a secondary electrode [70]. Hong et al. fabricated an elastic, fibrous, composite sheet with biodegradable poly(ester urethane) urea (PEUU) and poly(lactide-co-glycolide) (PLGA) using a two-stream electrospinning setup with a rotating metal rod as the collector [71]. Such composite sheets possessed better breaking strains, tensile strength and suture retention capacity. In a review article, Persano et al. discussed different multi-jet configurations and the further possibilities of syringe-free approaches to multi-jet electrospinning [45].

### 3.3. Collector Modification

The collector plate can be configured based on the application of polymeric fibers. Commercially, the most common collectors are stationary plates (or aluminum foil) and rotating plates. Both these types of collectors can be subdivided further into continual or patterned. A continual collector is simple and can be used to obtain fibers with a random internal structure on a stationary type, while some degree of directional control can be gained with the higher speed of a rotating collector. The patterned collectors have thin conductive wires separated from one another by an air gap. During the process, the fibers are deposited either between or perpendicular to the wires, individually, achieving some degree of alignment. Properly aligned polymer fibers are mostly useful in tissue engineering. In the rotating type of patterned collector, operation at lower speeds deposits fibers between the conductive wires, while deposits at the higher speed are dependent on electrostatic and mechanical forces increasing the degree of alignment. For research purposes, different designs of collectors have been used, including: mesh [72], pin [73], grids [74], liquid bath [75,76], rotating rods [77], rotating cylinder [78], parallel bars [77], rotating drum with wire wound on it [79], and disc [34,80].

## 4. Methods of Incorporating Drugs

Electrospinning is easy and cost effective, and it offers great flexibility in selecting materials, high loading capacity and high encapsulation efficiency, which makes it suitable for medical and drug-related research. There are various methods of drug loading in the polymeric solution for electrospinning.

### 4.1. Blending

Blending is the primary method for incorporating drugs into the polymer solution by dissolving or dispersing the drug and then subsequently electrospinning. Though this method is simple and easy, the physicochemical properties of the drug and the polymer need to be precisely considered, as these affect the encapsulation efficiency, the drug distribution in the fiber and the kinetics of the drug release. Lipophilic drugs (e.g., paclitaxel) should be dissolved in a lipophilic polymer and hydrophilic drugs (e.g., doxorubicin hydrochloride) in a hydrophilic polymer for better encapsulation. When the drug is not dissolved properly in the polymer solution, a dispersion is obtained, which might lead to burst release if the drug migrates to the fiber surface [81]. To obtain a sustained release of the drug from the electrospun fibers, in order to enhance the drug-loading efficiency and to reduce the burst release, different combinations of the mixtures of hydrophilic and hydrophobic polymers are used [82,83,84]. This process was modified by Ma et al. to obtain highly porous chitosan nanofibers by electrospinning chitosan/polyethylene oxide (PEO) blend solutions and then removing PEO with water [85]. They then soaked the porous nanofibers in 0.1 wt% paclitaxel solution to load the drug, and then into 4 wt% hyaluronic acid for encapsulation. Mickova et al. have compared electrospinning of a liposome by blending and by coaxial electrospinning and have reported that blend electrospinning could not conserve intact nanofibers [86].

### 4.2. Surface Modification

Surface modification is the technique in which the therapeutic agent is bound or conjugated to the fiber surface to make it structurally and biochemically similar to the tissue. The drug release in this case will be attenuated, and the functionality of the biomolecules will be protected [87]. The burst release and short-term release will be mitigated with this strategy, making it highly applicable for slow and prolonged delivery of gene or growth factors. Incorporation of DNA, growth factors and enzymes, conjugated to fibers, preserves their bioactivity and functionality [87,88,89,90]. The modulation of drug release can also be obtained if surface modification is done on blended electrospun fibers. Im et al. fluorinated the electrospun fibers to obtain controlled release of the drug by introducing a hydrophobic group onto the surface [91]. In one modification, Yun et al. oxyfluorinated the multi-walled carbon nanotubes (MWCNTs) to introduce the functional groups and improve the compatibility with the PVA/polyacrylic acid (PAA) polymer solution before electrospinning the composite mixture [92]. Kim et al. surface modified the fiber and loaded it with small-interfering RNA (siRNA) to obtain better results in gene silencing and wound healing [93]. Co-axial electrospinning encapsulates biomolecules like DNA into the fiber, in contrast to surface localization of DNA fibers with the blending process. Luu et al. reported that the transfection efficiency of electrospun DNA was significantly lower than Fugene 6, a commercially available transfection mediation agent [94]. To overcome the drawback, the electrospinning method was changed to coaxial to get a polymer coating around the biomolecules, which not only modified the release, but also protected the core against the direct exposure to the environment [60,62,95]. For additional benefits, the shell polymer can also be loaded with other bioactive molecules, such as non-viral gene-delivery vectors, for delivering the released DNA [62].

### 4.3. Emulsion

Another approach is the process of forming an emulsion for electrospinning, where the drug or the protein solution is emulsified within a polymer solution. The latter acts as an oil phase, and spinning such an emulsion produces a well-distributed fiber for a low molecular weight drug [96] and a core-shell for a high molecular weight drug [97,98,99]. The success of this process is mainly dependent on the ratio of the aqueous solution to the polymer solution. This governs the distribution behavior of the molecule in fiber, which in turn determines the release profile, structural stability and bioactivity of the encapsulated biomolecules [97]. As the drug and the polymers are dissolved in appropriate solvents, avoiding the need of a common solvent, various combinations of hydrophilic drugs and lipophilic polymer can be used. Unlike coaxial spinning, emulsion spinning might damage macromolecules, such as pDNA, due to shearing force and interfacial tension between the two phases. Such instances can be avoided by preventing denaturation by the condensation of pDNA [98,99].

### 4.4. Multi-Drug Delivery

**Figure 5:**
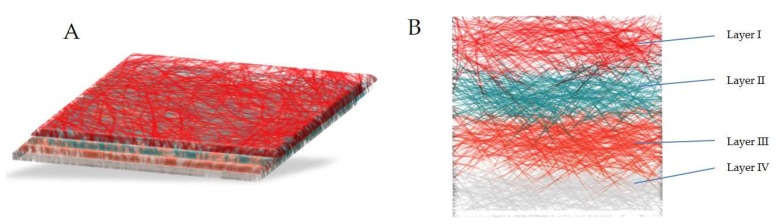
Graphical presentation of multi-drug delivery system: (**A**) overview; (**B**) cross sectional view of a tetra-layered sequential electrospun mesh. Cross-sectional view consists of drug loaded mesh (layer I), barrier mesh (layer II), second drug loaded mesh (layer III) and basement mesh (layer IV). Redrawn from [102].

Multi-drug delivery is a recent approach in which multiple drugs with or without similar therapeutic effects are combined and electrospun with suitable polymer(s) [100,101]. Wang et al. have used drug-loaded polymeric nanoparticles for the core and drug-loaded polymer for the sheath to obtain a chain-like structure with a distinct release behavior, enabling a program or temporality release of multiple agents [100]. Xu et al. have developed a hydrophilic model of bovine serum albumin (BSA) drug-loaded chitosan microspheres and suspended them in poly(l-lactic acid) (PLLA) solution with a hydrophobic model drug (benzoin) and polyvinylpyrrolidone (PVP) as a release tuner [101]. It is difficult to achieve independent release of the drugs in a multidrug system, as both drugs are held by the carrier, which provides the same diffusion pathways and matrix-degradation rate [95]. Okuda et al. have developed a multilayered drug-loaded electrospun nanofiber mesh fabricated for time-programmed dual release by sequential electrospinning. The formulation has four layers: the drug-loaded mesh (the top layer), the barrier mesh (blank polymer), the second drug-loaded mesh and the last basement mesh. This system provided for the development of electrospun fibers with controlled drug release and time of release by optimizing the fiber size, thickness of each layer and relative position of the layer. Though the authors have used two dyes as model drugs, this approach is significant for the biochemical modulation in chemotherapy with multiple-anti tumor drugs [102]. A graphical presentation of the same is shown in Figure 5.

### 4.5. Multilayer Coated

Yet another innovative method of incorporation and delivery of drug combines the large surface area of electrospun fibers with polyelectrolyte multilayer structures [95]. Such a multilayer uses either electrostatic or hydrogen bonding or acid-base pairing in layer-by-layer adsorption of polymers [103]. Chundar et al. have used two oppositely charged weak polyelectrolytes, polyacrylic acid (PAA) and poly allylamine hydrochloride (PAH), to produce nanofibers loaded with methylene blue as a model drug. In addition to polymers, a hydrophobic layer of perfluorosilane and PAA/poly(N-isopropylacrylamide) (PNIPAAM) was coated. The latter also provided temperature-controlled drug-release properties. Im et al. used alginate and chitosan to coat the lactobacillus-incorporated polyvinyl alcohol-based electrospun fibers. This formulation was designed to release lactobacillus in the large intestine after chitosan and alginate were dissolved in the acidic and then neutral environments of the GIT [104].

## 5. Electrospun Nanofibers in Drug Delivery

The electrospinning process has been used in drug delivery for treating various diseases. Electrospun fibers are administered mainly via oral and topical routes and as implantable systems.

### 5.1. Vitamins, NSAIDS and Natural Products

The transdermal drug delivery system (TDDS) delivers the drugs locally or systemically via the skin. This is mainly suitable for drugs that cannot be taken by the oral route, either because of extensive degradation in the GIT or because the drug undergoes extensive first pass metabolism [105]. Electrospun fibers of vitamins, anti-inflammatory and antioxidant drugs are mainly given by transdermal route [92,106,107,108,109,110,111]. Taepaiboon et al. have loaded vitamin A acid (all-*trans* retinoic acid) and vitamin E (α-tocopherol) in cellulose acetate polymer-based electrospun fibers and solvent cast films [110]. The results obtained revealed that the electrospun fiber mats showed a gradual and consistent increase in the cumulative vitamin release assayed using a total immersion technique over the test period of 24 h for vitamin E- and 6 h for vitamin A-loaded fiber. The corresponding solvent cast films showed a burst release of vitamins. A similar study performed by Nagwhirunpat et al. compared the electrospun and solvent cast film of polyvinyl alcohol loaded with meloxicam, an anti-arthritis drug [111]. They observed significantly higher skin permeation flux of the drug from electrospun fiber mats than with the solvent cast film, and the flux increased with an increase in drug concentration in both cases. Yun et al. developed an electro-responsive TDDS fiber by electrospinning poly(vinyl alcohol)/poly(acrylic acid)/multi-walled carbon nanotubes (PVA/PAA/MWCNT) with ketoprofen. The swelling, drug-release properties and conductivity of nanofibers were dependent upon the MWCNT, oxyfluorination and oxygen content during oxyfluorination [92]. The fibers were found to be non-toxic and biocompatible, with cell viability of more than 80%. In a similar study, Im et al. worked to understand the effect of MWCNT on ketoprofen delivery in an electro-sensitive TDDS with polyethylene oxide and pentaerythritol triacrylate polymers [109]. This study supported the impact of MWCNT in influencing conductivity and drug-release behavior. Reda et al. formulated ketoprofen-loaded Eudragit^®^ L and Eudragit^®^ S electrospun nanofibers for treating oral mucositis. The rapid evaporation of solvent during electrospinning prevented the drug molecules from forming crystalline aggregates within the nanofibers. In addition, the amount of ketoprofen released from nanofibers was significantly higher than that released from corresponding solvent-casted films. Furthermore, a marked reduction in inflammatory infiltrate was seen in mucositis-induced rabbits with developed nanofibers [112]. Apart from the synthetic chemicals, a natural/herbal extract containing asiaticoside from *Centella asiatica* was incorporated into the electrospun fibers based on cellulose acetate polymer [106]. Suwantong et al. used two forms of the drug, pure asiaticoside and a crude extract from the plant. They observed a better release profile from the pure drug than from the extract assayed using the immersion method, while the release from both fiber mats was significantly low in the pigskin method. In addition, they checked the release profile of the solvent cast films, which were much lower. They further reported that the extract-loaded fibers and films were toxic to normal human dermal fibroblasts at the extraction concentrations of 5 and 10 mg/mL [106].

### 5.2. Antibiotics/Antibacterial Agents and Wound Dressing

In recent years, antibiotics and antibacterial agents have been the most common drug molecules that are encapsulated, using different polymers and their combinations as carriers. Different polymers, such as PLA, PLGA and PCL, are primarily used in the polymeric electrospun fibers for biodegradability, and other natural and synesthetic hydrophilic or hydrophobic polymers are used to control the release pattern of the drug. Kenaway et al. used tetracycline hydrochloride with poly(ethylene-co-vinyl acetate) (PEVA), PLA and their 50:50 blend to deliver the drug for treating periodontal disease [113]. They reported over five days of release with PEVA and the blend, suggesting their applicability in controlled-release technology. In another study by Alhusein et al., electrospun fibers of tetracycline HCl using PCL and PEVA were developed for potential application in wound healing and skin-structure infections. The developed three-layered electrospun matrix showed controlled release and also higher antibacterial efficacy when compared to the commercially available test disks of the drug [114]. The same group of researchers also reported high biological activity of the developed fiber matrices in complex models of biofilm formation. The fibers killed preformed biofilms and mature dense colonies of *Staphylococcus aureus* MRSA252 and also inhibited the formation of new biofilms [115]. Wang et al. have electrospun PVA nanofibers containing pleurocidin, a novel, broad-spectrum antimicrobial peptide, for food preservation applications [116]. Direct application of the active moiety is not possible because of the loss in its bioactivity. This study reported higher inhibition efficacy of the drug from nanofibers against *Escherichia coli* in apple cider.

Wound dressings protect the wound from external microorganisms and absorb/adsorb the exudate from the wound, providing an acceptable cosmetic appearance. The use of different components in the wound dressing prevents infection of the wound and accelerates the healing process [117]. The components added to the inert dressings are mainly bioactives in the form of films, hydrogels, foams and sponges [84]. With respect to wound healing, electrospun fiber mats have the advantage of high surface area for efficient absorption of exudates; in addition to adjusting the moisture of the wound and promoting scar-free regeneration of skin cells, these mats have porosity enough to supply oxygen for cell respiration, yet not enough for bacterial infections [58,117,118]. In one study by Jannesari et al., electrospun nanofibers of poly(vinyl alcohol) and poly(vinyl acetate) were prepared individually and in a 50:50 blend of the polymers. The nanofibers prepared using the polymer blend sustained the drug release and were found to be comfortable due to significant swelling [84]. In an in vitro microbial study, Said et al. have shown a faster bacterial colonization and biofilm formation in fusidic acid-loaded PLGA fibers, which in turn enhanced the release of the drug, eradicated planktonic bacteria and suppressed the biofilm [119]. Thakur et al. used the dual spinneret electrospinning apparatus to prepare a single scaffold of lidocaine and mupirocin [120]. Two drugs with varying lipophilicities were found to have different release profiles. Lidocaine showed a burst release, while mupirocin showed sustained release, providing action for over 72 h. Wang et al. have fabricated nanofibers of ethylene-co-vinyl alcohol (EVOH) with different antibacterial drugs and silver for wound dressings with superior germ killing capacity [121]. Silver nanoparticles have been used in many other studies for similar applications [66,122]. Recently, Chutipakdeevong et al. have utilized the hybridization method to combine the properties of *Bombyx mori* silk fibroin with poly(ε-caprolactone) (PCL) electrospun fibers [123]. The PCL fiber surfaces were coated with silk fibroin protein using the lyophilization technique and then surface modified with fibronectin to improve their biological function. The surface-modified hybrid showed significant proliferation of normal human dermal fibroblast (NHDF), followed by hybrid scaffold and then neat PCL fibers.

### 5.3. Delivery of Anticancer Agents

Anticancer agents, such as doxorubicin, paclitaxel, cisplatin and dichloroacetate, have been incorporated into electrospun fibers with polymers such as PLA, PLGA and PLLA for postoperative chemotherapy. A water-in-oil (*w*/*o*) emulsion, with water soluble drugs in aqueous phase and polymeric solutions of PEG-PLA in chloroform as oily phase, was prepared and electrospun to obtain fibers [124]. In a continuation study, hydrophobic paclitaxel and hydrophilic doxorubicin were simultaneously loaded in the emulsion based electrospinning process for multi-drug delivery [125]. The cytotoxicity study of rat Glioma C6 cells showed higher inhibition and apoptosis in combination therapy compared to the single-drug system. In another study, an in vitro cytotoxicity study of the same cells showed a sustained release of platinum-based cisplatin for more than 75 days without burst release and four times better cytotoxicity than the free drug [126]. Lee et al. have fabricated biodegradable PLGA fibers sheets for local delivery of epigallocatechin-3-O-gallate (EGCG) to reduce intimal hyperplasia in injured abdominal aorta [116]. The EGCG-loaded sheets exhibited initial burst release for 24 h, followed by sustained release for more than 30 days in phosphate buffer. In vivo studies showed promising results against intimal hyperplasia after application of the EGCG-loaded fibers, compared to PLGA control.

Recently, electrospun fibers have been used for local chemotherapy. Liu et al. encapsulated doxorubicin in PLLA polymer fiber and examined its efficacy for local chemotherapy against secondary hepatic carcinoma by wrapping the whole liver with carcinoma with the fiber-mat [127]. In the first 24 h, the drug was rapidly released from the fiber to localize in the liver tissue. It also significantly inhibited the tumor growth and increased the median survival time of the mice in the experiment. Luo et al. prepared fibers with core-loaded hydroxycamptothecin (HCPT) and 2-hydroxypropyl-β-cyclodextrin complexed HCPT, and observed that the inclusion complex showed superior antitumor activity and fewer side effects compared to the free drug [128]. Ma et al. loaded paclitaxel in the porous chitosan nanofiber and then encapsulated the fiber in polyanionic macromolecular hyaluronic acid (HA). The MTT assay in prostate cancer cells showed that the drug-loaded nanofiber mats were good at prohibiting cell attachment and proliferation [129]. The drawback of the fibers was an initial burst release of drug within the first 48 h. Chen et al. incorporated titanocene dichloride in PLLA polymer-based fibers [130]. The MTT assay in human lung tumor (SPCA-1) cells showed that drug contents of 40, 80, 160 and 240 mg/L had cell growth inhibition rates of 11.2%, 22.1%, 44.2% and 68.2%, respectively. Apart from the synthetic anticancer drug, natural products with anticancer properties and minimal side effects have been studied. Suwantong et al. electrospun curcumin in cellulose acetate solution and observed that curcumin is almost completely (~90 to ~95%) released in the total immersion method, while considerably low values were obtained for transdermal diffusion through pig skin [108]. In another study, Shao et al. fabricated green tea polyphenols (GTP) in poly(ε-caprolactone)/multi-walled carbon nanotube (PCL/MWCNTs) composite nanofibers by electrospinning. The cytotoxicity experiment showed a significant inhibitory effect in A549 and Hep G2 tumor cells [131]. Table 1 provides a compilation of studies involving the use of electrospun fibers in delivering antibiotics, anticancer agents and NSAIDS.

### 5.4. DNA, RNA, Protein and Growth Factor Delivery

The most commonly loaded bioactive materials in electrospun fibers include DNA, RNA, proteins and growth factors. The electrospinning process applied to the bioactive materials should be designed in such a way that the activity and functional efficacy of the material is preserved during and after electrospinning. Before coaxial electrospinning, few studies on bioactive materials had been done with the blending process. The stability of the growth factors is the limiting aspect in formulating them in tissue-engineered scaffolds. Human nerve growth factor (hNGF) was encapsulated with BSA as a carrier protein into the nanofibers of PCL and poly(ethyl ethylene phosphate) (PCLEEP). The protein released in a sustained manner for more than three months from the electrospun fibers and showed partial retention of bioactivity [148]. The same group also co-encapsulated small-interfering RNA (siRNA) and transfection reagent (TKO) complexes within a nanofiber of caprolactone and ethyl ethylene phosphate (PCLEEP, diameter ~400 nm) to obtain a sustained release of siRNA for up to 28 days [149]. The release of siRNA was enhanced and more significant gene knockdown was obtained when compared with electrospun fibers of PCL containing siRNA [150]. Schneider et al. demonstrated that biofunctionalized silk mats containing epidermal growth factor (EGF) are extremely promising in achieving bioactive wound dressings for the wound healing process [151]. Similarly, Zhang et al. showed that the poly(ethylene carbonate-ε-caprolactone) scaffolds with VEGF maintained good growth and spread morphology in human umbilical vein endothelial cells [152]. After the introduction of the coaxial electrospinning method, most biomolecules are preferentially encapsulated using this method, forming a core of biomolecule and shell of polymer in a core-shell structure. The polymeric shell protected and released biomolecules in a sustained manner. Saraf et al. prepared fiber scaffolds with plasmid DNA (pDNA) within the core of poly(ethylene glycol) and non-viral gene delivery vector poly(ethylenimine)-hyaluronic acid (PEI-HA) within the sheath polymer poly(ε-caprolactone) (PCL) by coaxial electrospinning [62]. They achieved variable transfection activity over extended periods of time upon the release of pDNA and non-viral gene delivery vectors from electrospun fiber scaffolds. Mickova et al. have proposed the use of liposomes in the core of polyvinyl alcohol (PVA) and shell of PCL for protecting the enzymatic activity of horseradish peroxidase [86]. The encapsulated enzyme retained its activity because of the shielding effect of the lipid sphere of liposome. Chen et al. encapsulated chitosan/siRNA nanoparticles in PLGA by electrospinning to control the release behavior at different pH conditions. In addition, the encapsulated siRNA showed up to 50% enhanced green fluorescent protein (EGFP) gene silencing activity after 48 h of transfection in H1299 cells [143]. Surface functionalization of nanofibers was performed for MMP-2-siRNA (Matrix metalloproteinase) in linear polyethyleneimine (LPEI) coated nanofibers with various nitrogen/phosphate (N/P) ratios [93]. In an animal study for seven days, it was observed that the siRNA in these fibers increased the MMP-2 gene silencing effect and neo-collagen accumulation at the wound site. Fabrication of surface modified electrospun fibers containing growth factors conjugated with heparin or polysaccharides is becoming common [87,89,153]. A burst release of nerve growth factor was observed from electrospun scaffolds conjugated with chitosan/poly(vinyl alcohol) [89]. Basic fibroblast growth factor was incorporated in heparin containing a polyelectrolyte nanoparticle, and that was electrostatically adsorbed on the chitosan matrix to overcome the problem of burst release [87]. Han et al. fabricated a composite design containing poly(ethylene glycol)-poly-(ε-caprolactone) diacrylate (PEGPCL) hydrogels coupled with electrospun mats of poly(ε-caprolactone) to control the burst release and to extend the release duration of nerve growth factor [154]. The bioactivity of the growth factor was demonstrated by PC-12 cells’ neurite extension. Further, immobilization and delivery of the growth factor for the bone tissue engineering is discussed in the review by Chen and Lv [155]. In addition, electrospinning has extensively been used and reviewed for tissue scaffold engineering with or without any active moiety incorporated in the polymeric matrix. Table 2 provides a list of studies involving the use of electrospun fibers in delivering proteins, DNA, RNA and human factors.

### 5.5. Nanoparticle Impregnated Nanofibers

Electrospun nanofibers containing nanoparticles is another area of research that has been widely investigated for various applications, including surface-enhanced Raman scattering [156], antimicrobial packaging [157], dye-sensitized solar cells [158], food preservation [159], biowarfare decontamination [160], water treatment [161], high-performance gas sensing [162], and environmental remediation [163]. In drug delivery, these novel impregnated nanofibers are being explored in the areas of wound care, regenerative medicine, dental engineering, and for cancer treatment. In this section, we briefly summarized biomedical applications of nanoparticle impregnated nanofibers.

A study conducted by Lee et al. investigated the efficacy of chitosan (CTS) nanofibers containing silver nanoparticles for topical wound care. Silver nanoparticles were generated directly in the chitosan solution by chemical reduction using sodium borohydride. The nanoparticle solution obtained was then poured into sodium hydroxide resulting in the formation of CTS/AgNPs composite, which was further subjected to electrospinning to form nanofibers. The prepared fibers showed excellent antibacterial activity against *P. aeruginosa* and methicillin-resistant *S. aureus* when compared to the pure CTS nanofibers [164]. Similarly, Shi et al. prepared Ag/polyacrylonitrile (Ag/PAN) anti-bacterial nanofibers for use in implant scaffolds and biotextiles. Initially, PAN was dissolved in DMF and a known amount of AgNO_3_ was added to form the pre-electrospinning solution. The solution was then treated using helium atmospheric plasma to reduce AgNO_3_ into metallic silver nanoparticles. Finally, the solution obtained was electrospun to form nanofibers containing embedded Ag nanoparticles. SEM images revealed the smooth and continuous nature of the nanofibers. The nanofibers demonstrated a sustained release of silver ions and also showed higher antibacterial activity against Gram positive (*B. cereus*) and Gram negative bacteria (*E. coli*) when compared to the untreated nanofibers [165]. Another study conducted by Castro-Mayorga et al., investigated the antiviral properties of Ag nanoparticles in coated polyhydroxyalkonates. The films of poly(3-hydroxybutyrate-co-3-hydroxyvalerate) (PHBV) were developed by depositing a coat of electrospun fiber mat containing post-processed PHBV18/Ag nanoparticles over PHBV3 films formed by compression molding. Energy dispersive X-ray (EDX) analysis showed that Ag nanoparticles were homogeneously distributed into the coating and on the PHBV3/PHBV18 film. Moreover, cell culture analysis revealed no infectious feline calicivirus (FCV) recovery when treated with the prepared films, while murine norovirus (MNV) was decreased by 0.86 log [166].

Nie and Wang studied the complex of DNA and poly lactide-co-glycolide (PLGA)/hydroxyapatite (HAp) composite scaffolds fabricated by electrospinning for their use in bone tissue engineering. DNA was incorporated into the scaffolds in the form of naked DNA or DNA/chitosan nanoparticles before/after fiber fabrication. The results revealed that the scaffolds were non-woven and predominantly composed of PLGA with a dispersion of HAp nanoparticles. The addition of HAp nanoparticles showed an increase in the release rate of DNA from the scaffolds containing both naked and encapsulated DNA. Moreover, the scaffolds with encapsulated DNA/chitosan nanoparticles also showed a higher cell attachment, greater cell viability and desired transfection efficiency in human marrow stem cells (hMSCs) [167]. A similar study conducted by Tanaka and co-workers fabricated hydroxyapatite/PLA composite electrospun nanofibers for bone tissue engineering. The surface-modified HAp nanoparticles of stearic acid were dispersed uniformly in the PLA nanofibers and were evaluated for their mechanical strength. The authors concluded that the electrospun fibers showed higher strength when compared to the unmodified ones [168]. Table 3 provides a partial listing of studies involving the use of electrospun fibers in tissue engineering.

Liposome-enriched nanofibers were investigated for delivering and preserving horseradish peroxidase (HRP) enzymatic activity. Liposomes were prepared using soybean derived l-α-phosphatidylcholine encapsulating horseradish peroxidase (HRP) by the extrusion technique. This study compared the activity of HRP in nanofibers developed by coaxial or blend electrospinning with and without liposomes. Blend nanofibers were prepared by electrospinning unilamellar liposomes dispersed in an aqueous solution of PVA and nanofibers, while the coaxial nanofibers were developed using PVA-core/PCL-shell with embedded liposomes. The results indicate that the blending of liposomes could not conserve the intact liposomes, while the nanofibers obtained by coaxial electrospinning retained liposomes. In addition, the core/shell nanofibers not only preserved the activity of encapsulated HRP enzyme but also enhanced the proliferation of mesenchymal stem cells (MSC) [86].

Poly (l-lactic acid-co-ε-caprolactone) (PLACL) nanofibers containing magnesium oxide (MgO) nanoparticles in synergy with aloe vera (AV), curcumin (CUR) and β-cyclodextrin (β-CD) were prepared and evaluated for treating breast cancer. In vitro toxicity of electrospun nanofibers composed of PLACL; PLACL with AV; PLACL with AV and MgO; PLACL with AV, MgO and CUR; and PLACL with AV, MgO, and β-CD was assessed in MCF-7 cells using the MTT assay. The results showed that PLACL nanofibers containing CUR showed the greatest cell death among all other nanofibrous scaffolds. However, PLACL nanofibers without MgO nanoparticles exhibited greatest tensile strength [176]. Sperm-shaped microrobots were studied for targeting breast cancer cells in vitro using fabricated nanoparticles. The microrobots were fabricated using electrospinning by a solution composed of polystyrene, dimethylformamide and nanoparticles of iron oxide. Under the influence of an oscillating magnetic field, the robotic sperm could controllably take S-shaped, U-shaped, square paths and selectively target the MCF-7 cells. Moreover, the cell membrane was not damaged after penetration of the robotic sperm into the cancerous cells [177].

Electrospun nanofibers composed of poly (ɛ-caprolactone) (PCL) and zero valent zinc nanoparticles were investigated by Sezer et al. to promote neuroglial cell proliferation. Chemical characterization studies indicated that the nanoparticles did not interact chemically with the PCL matrix. In addition, the nanofibers enhanced the tensile strength of the PCL matrix and promoted cell proliferation depending on the amount of zinc nanoparticles embedded inside the PCL matrix [178]. Another study investigated the efficiency of electrospun hybrid scaffolds composed of cyclosporine-loaded PLGA nanoparticles embedded in PCL scaffolds for promoting innervation of bioengineered teeth. The results from histological studies showed that the implantation of designed scaffolds in adult ICR mice did not alter the development of teeth. In addition, the transmission electron microscopy (TEM) and indirect immunofluorescence studies showed that 88% of the teeth were innervated upon treating with designed hybrid scaffolds [179].

## 6. Commercialization Challenges of Electrospinning

Although the benefits of electrospinning have been largely demonstrated in various fields of science, there is still a great need to implement the production in an efficient way. Several challenges relating to the electrospinning process are yet to be addressed. These include: (a) large-scale manufacturing; (b) accuracy and reproducibility during all the fabrication steps; and, (c) safety and environmental aspects of electrospinning [45]. The major challenges involved in mass production of electrospun fibers include low output per spinneret, clogging of the spinneret tip, inter-jet interference, recovery of vaporized solvents involved in the process, and fiber alignment over a large area of substantial thickness. To produce electrospun nanofibers without any morphology defects, the solution concentration is kept to a minimum, with solvent making up more than 70% of the solution mass. Therefore, only a fraction of the solution passing through the spinneret actually contributes to the mass of the nanofiber produced [180]. Also, there is a limit on the feed-rate per nozzle and higher rates might result in dripping of the solution from the nozzle, especially if the nozzle is placed in the middle. This is due to the insufficient electric field experienced by the nozzles, which results in inadequate drawing of the solution.

Clogging of the spinneret due to the solution gelation is highly disruptive and causes production losses. This problem is more apparent with solutions of higher concentration, as they are more viscous [181] and with polymers of high degree of crystallinity [182]. In addition, a solvent with low boiling point also causes clogging of the spinneret tip [183]. While the productivity of the microfiber spinning process can be increased by placing many spinnerets per spinning head, this is not feasible in the case of electrospinning. In electrospinning, the ejected solution spreads out to form an expanding cone and it interferes with the neighboring jets if the spinnerets are packed too close to each other [68]. Uniformity in the fiber layer thickness deposited onto substrate material is also comprised by the inter-jet interference [180].

Another important concern is regarding the solvent used in the electrospinning process. This particular issue is highly important not only for safety reasons during fabrication, but also for the final products, as solvent residues might be trapped inside the electrospun nanofibers. Accurate control over solvent residues becomes crucial in the case of large-volume, solvent-based electrospinning for biomedical and pharmaceutical applications [45]. However, the use of solvent-free spinning should eliminate the risk of solvent residues and recovery of solvents [184]. Due to the lack of reliable and affordable electrospinning technologies, the use of many different active polymeric materials for the fabrication of nanofibers is still limited [45]. In tissue engineering, the other main challenges that impede the progress of electrospinning applications are increasing scaffold thickness and pore size. Moreover, it is highly difficult to produce identical scaffolds, especially between research groups, which narrows the use of electrospun fiber mats for tissue engineering applications. Also, with tailored electrospun fibers, it is challenging to ensure uniformity of the fibers with specific morphologies and properties [185].

The recent development of needleless electrospinning offers the possibility for fabricating electrospun nanofibers on a large scale and addresses the issue of clogging at the spinnerets [186]. Needleless electrospinning systems using rotating disks [187], rollers [188], balls [189], and bubbles [190] that produce huge amounts of nanofibers have been reported by a few researchers. This method involves the formation of numerous small droplets on the drum surface or disk/coil. Moreover, this method does not require the maintenance of Taylor cones throughout the process making it highly advantageous over conventional needle-based method. Nonetheless, this process consumes a lot of energy in order to maintain the high voltage required and thus increases the chance of spark generation in the system. Therefore, solvents with low flash points, such as chloroform, tetrahydrofuran (THF), and toluene cannot be used for needless electrospinning [182]. Another area where considerable progress has been made in recent years is the development of smart electrospun nanofibers. These nanofibers respond to various kinds of stimuli, including pH, ionic strength, temperature, light, electricity, and magnetic field, and undergo physical and/or chemical changes. Studies involving smart electrospun nanofibers, their advantages and shortcomings are detailed in recent review by Weng and Xie [191].

## 7. Conclusions

The electrospinning process has generated a lot of interest in various medical applications due to its ease of use, adaptability and flexibility in controlling the fiber diameter from the micrometer down to the nanometer range. Though this method has been in use for a few decades now, the techniques and the equipment used in the electrospinning process are ever-evolving. Electrospinning began with a single nozzle configuration and evolved into multi-nozzle configurations through coaxial and emulsion spinning configurations. Further studies are being carried out to modify the nozzle configuration and collector design in order to significantly improve fiber properties and simplify the manufacturing process. This review summarizes several key aspects of electrospinning in the use of electrospun fibers in drug delivery with a special emphasis on electrospun nanofibers impregnated with nanoparticles. Studies involving biomedical applications of electrospun nanofibers are also discussed. By careful selection of polymers, it is now possible to deliver various antibacterial agents and anticancer drugs in a required manner using electrospun nanofibers. In order to make further progress, particularly in the field of drug delivery, it is necessary to identify ways that allow large-scale fabrication of nanofibers with desired morphological and mechanical properties in a reproducible manner. Despite the relentless efforts being made by academic and industrial scientists, much of the research conducted with electrospun fibers is in vitro. Further progress in the field of electrospun nanofibers will require continued assessment in vivo. Scientists working in this field have to identify ways to use nanofibers for immunotherapy, gene therapy and regenerative medicine in order to meet the current, as well as any future, demands in drug delivery.

## Figures and Tables

**Table 1:** Studies involving the use of electrospun fibers in drug delivery (partial listing).

Drug(s)	Polymer(s)	Solvent Composition	Spraying Type
**Antibacterial agents**			
Tetracycline hydrochloride	PEUU and PLGA [71]; PLA, PEV, PLA/PEVA [113]; PLA/PCL [132] PLLA [133,134]; PCL, PEVA [114]	1,1,1,3,3,3-Hexafluoro-2-propanol [71]; Chloroform [113]; Chloroform, dimethylformamide [132]; Chloroform: acetone (2:1) [133,134]; chloroform:methanol (9:1) [114]	Single nozzle; Coaxial [133,134]
Gentamycin sulfate and Resveratrol (antioxidant)	PCL [133]	Chloroform: ethanol (3:1)	Coaxial
Ciprofloxacin Hydrochloride	PVA, Poly(vinyl acetate) [84]	Diluted acetic acid solution	Single nozzle
Fusidic acid and rifampicin	PLGA [135]	Tetrahydro Furan/Dimethylformamide	Single nozzle
Mefoxin	PLGA [83]	DMF	Single nozzle
Metronidazole benzoate	PCL [136]	Dichloromethane (DCM:DMF)	Single nozzle
Ciprofloxacin hydrochloride, Levofloxacin hemihydrate, Moxifloxacin hydrochloride	coPLA, coPLA/PEG [137]	DCM:DMSO (3:1)	Single nozzle
Lidocaine and mupirocin	PLLA [120]	Hexafluoroisopropanol	Dual spinneret
Ornidazole (Biteral^®^)	PCL	Chloroform and DMF (3:7)	Single nozzle
Potassium 5-nitro-8-quinolinolate	Chitosan/PEO [138]	2% (*w*/*v*) acetic acid	Single nozzle
Itraconazole and ketanserin	PU [139]	DMF, DMAc	Single nozzle
Pleurocidin	PVA [116]	Distilled water	Single nozzle
**NSAIDS**			
Ketoprofen	PVA/PAA/MWCNT [92]; PEO/PETA/MWCNT [109]; EC and PVP [140]; PVP/Zein [141]	Deionized water [92,109] Ethanol-water [140,141]	Single nozzle Coaxial [141]
Ibuprofen	PLGA PEG-g-CHN [142]	DMF	Side-by-side
Fenbufen	PLGA/Gelatin [82]	2,2,2-trifluoroethanol	Single nozzle
Rhodamine B/Naproxen	Chitosan nanoparticles/PCL composite [100]	Acetic acid/chloroform: methanol (3:1)	Single nozzle yet core/sheath fiber
Meloxicam	PVA [111]	Water	Single nozzle
**Anticancer agents**			
Doxorubicin [127] Doxorubicin Hydrochloride	PLLA [127] PEG-PLA [124,143]	Chloroform-methano-DMSO [127] Chloroform [143] w/o emulsion [124]	Single nozzle Emulsion [124]
Hydroxycamptothecin	HPCD [128]	DMSO	Emulsion
Paclitaxel	Chitosan/PEO/HA [84] PLGA [144,145]	Acetic acid/distilled water [84] DCM and DMF	Single nozzle
Cisplatin	PLA/PLGA [126]	DCM	Single nozzle
Dichloroacetate	PLA [146]	Chloroform	Single nozzle
1,3-Bis(2-chloroethyl)-1-nitrosourea	PEO and PEG-PLLA [147]	Chloroform	Single nozzle/Emulsion
Curcumin	Cellulose acetate [108]	Acetone/dimethylacetamide (2:1)	Single nozzle
Green tea polyphenols (GTP)	PCL/MWCNT [131]	Dichloromethane	Single nozzle
Titanocene dichloride	PLLA [130]	Dichloromethane	Single nozzle

PLA: Poly(lactic acid); PEVA-Poly(ethylene-co-vinylacetate); PCL: poly(ε-caprolactone); PCL-co-PCLEEP: Copolymer of caprolactone and ethyl ethylene phosphate; PLGA: Poly(d,l-Lactic acid-co-glycolic acid); PEUU: poly(ester urethane) urea; EC: ethyl cellulose; PEO: Poly(ethylene oxide); PU: Polyurethane; DMAc: Dimethylacetamide; PETA: pentaerythritol triacrylate; PEI-HA: Poly(ethylenimine)-hyaluronic acid; PVP: polyvinylpyrrolidone; PEG-g-CHN: poly(ethylene glycol)-g-Chitosan; PEG-PLA: poly(ethylene glycol)-poly(lactic acid); PLLA: poly(l-lactic acid); HPCD: 2-hydroxypropyl-β-cyclodextrin; HA: Hyaluronic acid; MWCNT: multi-walled carbon nanotubes, PAA–Poly(acrylic acid); PECCL: poly(ethylene carbonate-ε-caprolactone); PDLLA: poly(d,l-lactic acid).

**Table 2:** Studies involving the use of electrospun fibers in delivery of proteins, DNA, RNA and human factors (partial listing).

Drug(s)	Polymer(s)	Solvent Composition	Spraying Type
plasmid DNA (pDNA)	PEI-HA [62]		Coaxial
siRNA	PCL [150]; PCLEEP [149] Chitosan/PLGA [169]	2,2,2-Trifluoroethanol (TFE) [150]; RNase-free water [149]; Hexafluoro-2-isopropanol/water [169]	Single nozzle
Human glial cell-derived neurotrophic factor	PCL-co-PCLEEP [170]	Dichloromethane	Single nozzle
Human β-nerve growth factor	PCL-co-PCLEEP [148]	Dichloromethane	Single nozzle
Endothelial growth factor VEGF	PECCL [152]	-	Single nozzle
Bovine Serum Albumin (BSA)	PEO [171]	Deionized water	Single nozzle
Lysozyme	PLA [97]	Chloroform	Emulsion
Human-nerve growth factor (NGF)	Poly(L-lactide-co-caprolactone) [172]	Chloroform	Emulsion
DNA	PLA-PEG and PLGA [94]	DMF	Single nozzle
Growth factors (VEGF, PDGF)	Poly(urethane) [63]	Chloroform: ethanol (75:25)	Coaxial
Horseradish peroxidase	PVA/PCL [86]	-	Coaxial

**Table 3:** Studies involving the use of electrospun fibers in tissue engineering (partial listing).

Drug(s)	Polymer(s)	Solvent Composition	Spraying Type
Wound healing, tissue engineering, hemostatic agent	Collagen-PEO [173,174]	Hydrochloric acid	Single nozzle
Adenovirus with gene for green fluorescent protein	Poly(ε-caprolactone) [60]	Chloroform: ethanol (75:25)	Coaxial
Guided tissue regeneration	PDLLA/PLGA [175]	Chloroform:DMF (9:1) and THF/DMF (3:1)	Single nozzle
